# Understanding of sub-band gap absorption of femtosecond-laser sulfur hyperdoped silicon using synchrotron-based techniques

**DOI:** 10.1038/srep11466

**Published:** 2015-06-22

**Authors:** Mukta V. Limaye, S. C. Chen, C. Y. Lee, L. Y. Chen, Shashi B. Singh, Y. C. Shao, Y. F. Wang, S. H. Hsieh, H. C. Hsueh, J. W. Chiou, C. H. Chen, L. Y. Jang, C. L. Cheng, W. F. Pong, Y. F. Hu

**Affiliations:** 1 Department of Physics, Tamkang University, Tamsui 251, Taiwan; 2 Department of Physics, Indian Institute of Science Education and Research, Bhopal 462066, India; 3 Department of Applied Physics, National University of Kaohsiung, Kaohsiung 811, Taiwan; 4 National Synchrotron Radiation Research Center, Hsinchu 300, Taiwan; 5 Department of Physics, National Dong Hwa University, Hualien 974, Taiwan; 6 Canadian Light Source Inc., Saskatoon SK S7N OX4, Canada

## Abstract

The correlation between sub-band gap absorption and the chemical states and electronic and atomic structures of S-hyperdoped Si have been extensively studied, using synchrotron-based x-ray photoelectron spectroscopy (XPS), x-ray absorption near-edge spectroscopy (XANES), extended x-ray absorption fine structure (EXAFS), valence-band photoemission spectroscopy (VB-PES) and first-principles calculation. S 2*p* XPS spectra reveal that the S-hyperdoped Si with the greatest (~87%) sub-band gap absorption contains the highest concentration of S^2−^ (monosulfide) species. Annealing S-hyperdoped Si reduces the sub-band gap absorptance and the concentration of S^2−^ species, but significantly increases the concentration of larger S clusters [polysulfides (S_n_^2−^, n > 2)]. The Si *K*-edge XANES spectra show that S hyperdoping in Si increases (decreased) the occupied (unoccupied) electronic density of states at/above the conduction-band-minimum. VB-PES spectra evidently reveal that the S-dopants not only form an impurity band deep within the band gap, giving rise to the sub-band gap absorption, but also cause the insulator-to-metal transition in S-hyperdoped Si samples. Based on the experimental results and the calculations by density functional theory, the chemical state of the S species and the formation of the S-dopant states in the band gap of Si are critical in determining the sub-band gap absorptance of hyperdoped Si samples.

Silicon (band gap *E*_g_ = 1.12 eV) that is hyperdoped with chalcogens (S, Se or Te) beyond the equilibrium solubility limit exhibits sub-band gap light absorption, making it a suitable material for Si-based infrared (IR) and photovoltaic applications^1^,^2^,^3^,^4^,^5^. In earlier studies it was claimed that above a critical concentration of dopants in Si, an insulator-to-metal transition (IMT) is caused by the formation of an intermediate band of the dopant states within the band gap of Si. Optically sensitive dopant states relatively deep in the band gap of the Si are generally believed to be responsible for the significant increase in the IR absorption. Such dopant states have been accepted as the origin of non-radiative recombination, facilitating the absorption of photons with energy that is less than the band gap of the Si. Previous investigations reported that sub-band gap absorption can be deactivated and reactivated through thermal treatment with laser irradiation^3^,^4^. The dopant diffusion causes the deactivation of sub-band gap absorption after thermal annealing and stabilization of high concentration of point defects/dopant states at high temperatures responsible for the reactivation. This phenomenon can be explained briefly in terms of diffusion theory^6^,^7^. The dopants contribute to IR absorption because they are present within crystalline grains and are coordinated with the Si lattices, consequently precipitating as a non-optically sensitive species^6^,^7^. Recently, Newman *et al*.^8^ performed Se *K*-edge extended x-ray absorption fine structure (EXAFS) studies of Se-hyperdoped Si samples and found that during annealing, the Se precipitated at grain boundaries and formed non-IR absorbing silicon diselenide (SiSe_2_) precipitates, reducing the sub-band gap absorption. The results of an EXAFS study revealed that thermal treatment facilitated the return of Se-hyperdoped Si samples to equilibrium atomic structural order with a reduction of the sub-band gap optical absorption. An Si *L*_3,2_ x-ray emission spectroscopic (XES) study of S-doped Si samples [up to 0.7 atomic percentage (at.%)]^9^ exhibited induced emission feature intensity above the Si valence-band-maximum (*E*_VBM_), which scaled linearly with S concentration associated with the S-dopant, and the line-shape of the S-dopant feature changed across the IMT. The annealing of the S-doped Si samples reduced the induced emission intensity, which was associated with the quenching of sub-band gap absorption^9^. Theoretical calculations suggested that the geometries and coordinations of the S impurities in clusters change upon annealing, altering the electronic and atomic structures and quenching sub-band gap absorption^10^. Density Function Theory (DFT) suggested that the chalcogen-induced IMT arises from the merging of dopant states and conduction bands in chalcogens hyperdoped Si^4^,^5^,^11^.

To understand better the optical properties that are relevant to IR or photovoltaic applications and to elucidate the physics of the IMT in Si-based hyperdoped with chalcogens, the relationship between the sub-band gap absorption and electronic and atomic structures need to be studied. Although many of investigations have focused on the optical properties of hyperdoped Si, the electronic and atomic structures of chalcogen dopants and the Si host, respectively, and their optical activity in relation to sub-band gap absorption in hyperdoped Si remain unclear.

This study investigates S-hyperdoped Si samples using synchrotron-based techniques such as x-ray photoelectron spectroscopy (XPS), x-ray absorption near-edge spectroscopy (XANES), EXAFS, valence-band photoemission spectroscopy (VB-PES) and first-principles DFT calculations. The XPS reveals that a high concentration of S, particularly as S^2−^ (monosulfide) species, in an absorbing sample with a high (~87%) sub-band gap and thermal annealing, increases the formation of non-IR absorbing larger S clusters [polysulfides (S_n_^2−^, n > 2)]. The results of XANES and VB-PES reveal that doping with S modifies the electronic structures of the hyperdoped Si samples. A Si *K*-edge EXAFS study further shows that S-hyperdoping induces high structural disorder around Si atoms. However, the nearest-neighbor (NN) bond length of Si-Si remains constant as the doping concentration of S is varied. There is no clear evidence that thermal annealing at 500 °C and 700 °C yields Si-S precipitates, presumably owing to the decrease in the sub-band gap absorption in chalcogen hyperdoped Si samples. Based on our experimental measurements, the chemical state of the S species and the S-dopant states in the band gap of Si are critical in determining the sub-band gap absorption of hyperdoped Si samples. A first-principles calculations using DFT method supports experimental results as well as the occurrence of the IMT at/above a critical concentration of S dopants in the hyperdoped Si samples. Our study provides clear evidence of the active chemical state of S species on the surface of Si and S-dopant states deep in the band gap of Si, are responsible for the sub-band gap absorption in the hyperdoped Si samples.

## Results and Discussion

**Figure 1:**
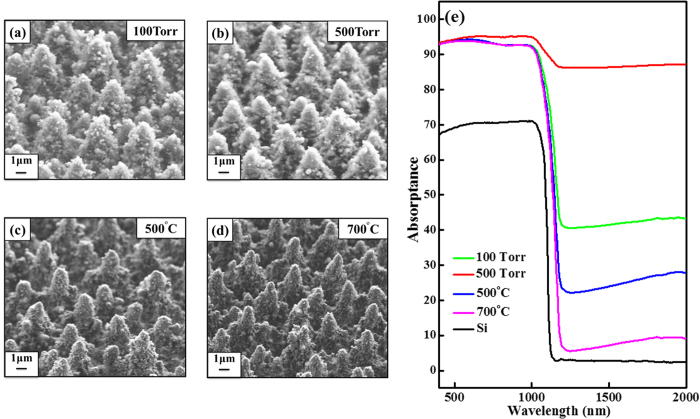
(**a**–**d**) SEM images of S-hyperdoped Si samples 100 Torr, 500 Torr, and the latter annealed 500 °C and 700 °C, respectively. (**e**) IR absorptance of S-hyperdoped Si samples.

Figure 1(a)–(d) present scanning electron microscope (SEM) images of the surfaces of the S-hyperdoped Si samples that were formed by femtosecond (fs) laser irradiation and annealing. The surface morphology of the S-hyperdoped Si samples varied from smooth to needle-like structure as the pressure of the SF_6_ gas increased. The needle-like microstructure of the samples prepared with a gas pressure of 500 Torr became increasingly porous as the annealing temperature increased from 500 °C to 700 °C. Figure 1(e) displays the IR absorption of S-hyperdoped Si samples and undoped Si(100) for reference. All S-hyperdoped Si samples exhibit broad and featureless absorption which is consistent with the results reported in the literature^6^,^7^,^9^,^12^,^13^. Clearly, the 100 Torr sample shows an average IR absorptance of ~42%, while that of the 500 Torr sample was much greater, ~87%. However, annealing the 500 Torr sample at 500 °C reduces the IR absorptance to ~23%, and annealing at 700 °C reduces it further to ~7%. Interestingly, the average IR absorptance of the sample that was annealed at 700 °C was closer to that of reference Si(100) than those of other hyperdoped Si samples. These results reflect the fact that the sub-band gap absorption of the hyperdoped Si samples was strongly related to concentration of S dopant and also affected by thermal annealing. Detailed investigations of the morphology and optical properties of hyperdoped Si samples, as effect of SF_6_ gas pressure and annealing temperature, can be found elsewhere^12^,^13^,^14^,^15^,^16^.

**Figure 2:**
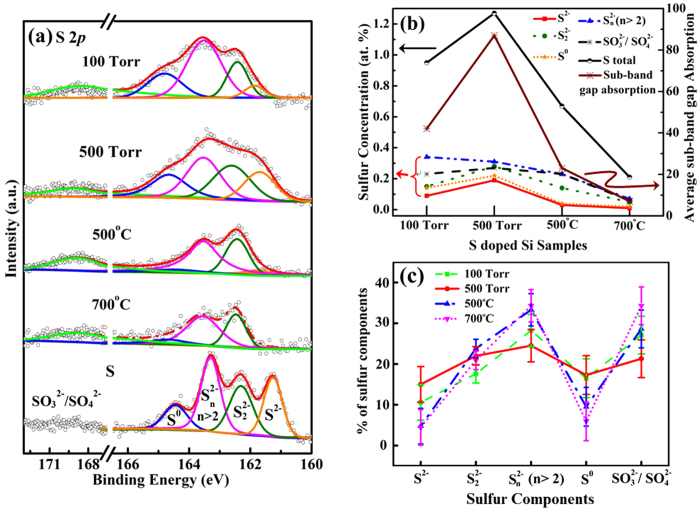
(**a**) Core-level XPS spectra of S 2*p* of hyperdoped Si samples, and for comparison, a standard sample of pure S. (**b**) Plot of S at.% and average sub-band gap absorptance in hyperdoped Si samples. (**c**) Plot of percentage of each S component in individual hyperdoped Si samples.

Figure 2(a) presents the S 2*p* core-level XPS spectra of S-hyperdoped Si samples prepared at 100 Torr and 500 Torr, and the 500 Torr sample that was further annealed at 500 °C and 700 °C, respectively. The S 2*p* core-level XPS spectrum of powder S is included for comparison. The XPS spectra of all the samples were deconvoluted into several features and Table SI of the Supplementary Information present detailed parameters such as energy position, full width at half maximum (FWHM) of the features and the percentage of each corresponding component. The at.% of each S species on the sample surface is obtained by dividing the integrated area under a characteristic feature by the total area under their corresponding core-level XPS spectra. The binding energies (BE) of S^2−^ (monosulfide), S_2_^2−^ (disulfide), S_n_^2−^ (n > 2, polysulfides) and S^0^ (elemental S) are located at ~161.2 eV, 162.3 eV, 163.3 eV and 164.4 eV, respectively^17^,^18^,^19^,^20^,^21^. In the spectra of the hyperdoped Si samples, one extra broad and weak feature at ~169–170 eV was observed and attributed to SO_3_^2−^ (sulfite) and/or SO_4_^2−^ (sulfate)^20^. Overall, as presented in Fig. 2(a), the general line-shape of S 2*p* spectrum of the S-hyperdoped Si samples is shifted to a higher BE (0.3–0.5 eV) than that of pure S and their characteristic features are broader, owing to a change in the chemical environment of the S-dopant in the Si matrix. Also, S species in the sample have more chemically inequivalent atoms than the reference S^22^. Clearly, as shown in Tab. SI, the 500 Torr sample yield a stronger S^2−^ (monosulfide) feature as compared to the 100 Torr sample. On the other hand, the annealed 500 Torr samples (annealed at 500 °C or 700 °C) have barely visible S^2−^ (monosulfide) feature [Fig. 2(a) & Tab. SI]. Annealing reduces the overall XPS intensity of the S 2*p* core-level features, revealing a lower concentration of S species on the surfaces of the annealed samples than those of the 100 Torr and 500 Torr samples. To calculate total at.% of S on the surface of each sample, XPS survey scans (in Fig. S1 of Supplementary Information) were performed and presented in the Tab. SI of the Supplementary Information. Although the sample surface was cleaned using HF to remove the surface oxides or carbon-related compounds before the synchrotron measurements were made, a considerable amount of O/C and small amount of N were observed in survey scan (Fig. S1 and Tab. SII of the Supplementary Information). The total at.% of the S 2*p* feature on the surface of the 100 Torr, 500 Torr and annealed samples (annealed at temperatures of 500 °C and 700 °C) were ~0.95%, 1.27%, 0.67% and 0.20% (Tab. SI), respectively. The surface XPS analysis revealed that the 500 Torr sample had a higher concentration of S than the other three S-hyperdoped Si samples. The observed diminution in the S concentration upon annealing of the 500 Torr sample (at 500 °C and 700 °C) is attributed to the diffusion of S atoms deep into the Si matrix as a result of thermal annealing^6^,^15^.

To obtain a correlation between the optical properties and the concentration of the S-dopant in the S-hyperdoped Si samples we have plotted the average sub-band gap absorption and the S at.% of the S-hyperdoped Si samples, shown in Fig. 2(b). The 500 Torr sample, which had the highest total S at.% (1.27%), exhibited the highest sub-band gap absorptance ~87%. The average sub-band gap absorptance reached ~42% in the 100 Torr sample, which contained 0.95% S in total. Annealing the 500 Torr sample at 500 °C and 700 °C drastically deactivate their absorptances to ~23% and 7%, corresponding S total at.% value 0.67% and 0.20%, respectively. As mentioned above, previous studies have attributed the deactivation of sub-band gap absorptance upon thermal annealing to the diffusion of dopants^6^,^15^. Those studies found that the sub-band gap absorptance of the samples decreases as the diffusion length of the dopant in the Si matrix increases. The diffusion lengths of S in Si samples which annealed at 500 °C and 700 ^o^C were ~30 nm and 250 nm, respectively. [The diffusion length of S dopant was calculated using diffusion length d = √Dt, where t is the annealing time; D is the bulk diffusivity of S dopant in the Si matrix, given by D = D_0_exp(−*E*_a_/*k*T); *k* is Boltzmann’s constant; T is the annealing temperature; and *E*_a_ and D_0_ are temperature-independent constants that were obtained from the literature on the bulk diffusivity of dopants]^6^. According to the analysis of S 2*p* core-level XPS results [Fig. 2(b)], the 500 Torr sample contains more S at.% than other S-hyperdoped Si samples. To understand further how the percentages of S in various chemical states varies among the samples and the effect of the S species in the sub-band gap absorptance, Fig. 2(c) presents the percentage of each S species in S-hyperdoped Si samples by normalization of total S concentration 100 on a scale in each corresponding samples. In the 100 Torr sample, the concentrations of the S^2−^ and S_2_^2−^ species are 9.1% and 15.4%, respectively and that of the S_n_^2−^ species is 36.0%. Notably, that S^0^ and SO_3_^2−^/SO_4_^2−^ species generally do not absorb energy in the IR range^23^,^24^. The 500 Torr sample contains 15.1% S^2−^, 21.8% S_2_^2−^ and 24.6% S_n_^2−^ species. Annealing of the 500 Torr sample at 500 °C (700 °C) reduced the concentrations of the S^2−^ and S_2_^2−^ species to 4.6% (2.3%) and 20.8% (22.7%), respectively, but increased the concentration of S_n_^2−^ to 34.5% (36.6%). Interestingly, the difference between the percentages of S^2−^ and S_n_^2−^ species is ~10% in 500 Torr, 17% in 100 Torr and much larger ~30% in the annealed samples. The percentage of S^2−^ species is higher in the 500 Torr sample than in the other S-hyperdoped Si samples. However, annealing the 500 Torr sample at 500 °C or 700 °C considerably increases the number of larger S clusters (S_n_^2−^) in relative to that of 500 Torr sample. However, annealing does not affect much the S_2_^2−^ species concentration. Mo *et al*.^10^ theoretically proposed that the formation of large clusters of dopant makes Si optically transparent, but S dimer species remain optically absorbing; they also claimed that in Si matrix S dopants are preferentially present at substitution sites and so S-dopant may introduce electronic states deep inside the band gap of Si, making the sample light-absorbing. Annealing of the samples increase the clusters of S species as S_n_^2−^ (n > 2), changing the optical properties as the geometry and coordination of S atoms get modified in the clusters. The results of the S 2*p* core-level XPS spectra provide clear evidence that the 500 Torr sample with the largest (~87%) sub-band gap absorption contains the highest concentration of S-dopant, especially S^2−^ species, and that thermal annealing increases the percentage of larger clusters S_n_^2−^ relative to that of S_2_^2−^ (Tab. SI), reducing the sub-band gap absorptance to 23% and 7% for the 500 °C and 700 °C samples, respectively. Therefore, a clear relationship is obtained between the chemical states of the S species and the sub-band gap absorptance of the S-hyperdoped Si. The classification of the IR-absorbing S species reveals that S^2−^ are the optically active species and thermal annealing increases the number of non-IR absorbing larger S clusters (S_n_^2−^, n > 2) in S-hyperdoped Si samples. The S_2_^2−^ species remain optically absorbing and concentration of these species does not change much after thermal annealing^10^.

Figure S2 in Supplementary Information presents the Si 2*p* core-level XPS spectra of S-hyperdoped Si samples. The Si 2*p* core-level XPS spectra of all the samples are deconvoluted, and the related parameters are presented in detail in Tab. SIII of the Supplementary Information^25^,^26^,^27^,^28^,^29^. Features that are associated with various O-containing Si species, Si_2_O (Si^1+^), Si_2_O_3_ (Si^3+^) and SiO_2_ (Si^4+^), were observed as presented in Fig. S2 and Tab. SIII. The Si 2*p*_3/2_ features of the 100 Torr and 500 Torr samples were shifted to a lower BE (at 99.3–99.4 eV) than that of the reference Si(100) (at 99.6 eV) and the shift was reversed by annealing. The observed feature shift (0.2–0.3 eV) is attributed to the structural disordering by presence of amorphous Si at the surface^29^,^30^. Micro-Raman spectroscopy verified the pressure-induced formation of Si polymorphs, as presented in Fig. S3 of the Supplementary Information. This result is consistent with previously made micro-Raman measurements on fs-laser-irradiated Si as the effect of pressure, indicating the formation of pressure-induced amorphous Si and/or Si polymorphs on the sample surface^31^ and they are recovered close to that of reference Si(100) upon annealing at high temperature.

**Figure 3:**
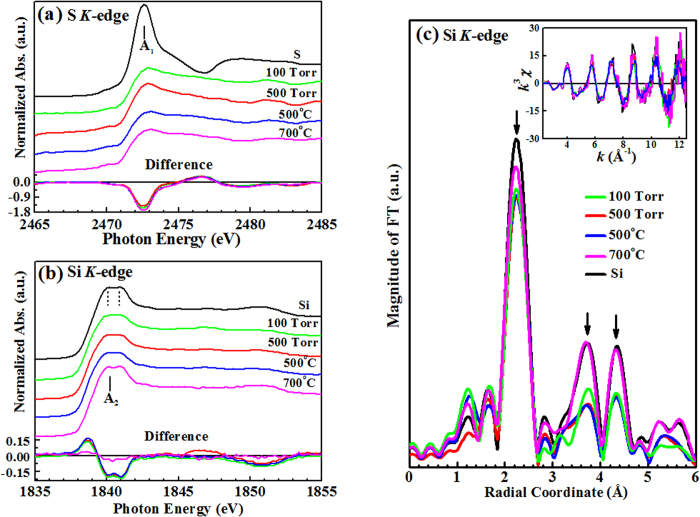
(**a**) S *K*-edge XANES of hyperdoped Si samples and pure S as a reference. Bottom panel shows the difference spectra of hyperdoped Si samples and reference pure S. (**b**) Si *K*-edge XANES of hyperdoped Si samples and reference undoped Si(100). Bottom panel shows the difference spectra of hyperdoped Si samples and reference Si(100). (**c**) FT of Si *K*-edge EXAFS spectra of hyperdoped Si samples for *k* between 2.3 and 12.5 Å^−1^. Inset plots EXAFS *k*^3^χ data.

Figure 3(a) displays the S *K*-edge XANES of the S-hyperdoped Si samples and pure S as a reference. According to the dipole-selection transition rule, the S *K*-edge XANES spectrum arise from the transitions of S 1*s* core electrons to unoccupied density of states (DOSs) with 3*p* character above the Fermi level. The line-shape of the S *K*-edge XANES spectra in the region 2472–2480 eV (denoted as A_1_) is broadened and the feature of the S-hyperdoped Si samples reduce gradually with incident photon energy, varying quite differently from that of pure S, which was sharp and intense. The lower panel in Fig. 3(a) shows the difference between the S *K* near-edge absorption spectra of S-hyperdoped Si samples and reference pure S. The intensity of feature A_1_ of the 100 Torr, 500 Torr and annealed samples (that are at 500 °C or 700 °C) is lower than that of reference pure S. The chemical states of the S species in S-hyperdoped Si samples are responsible for the broad and declining feature, which is unlike that of pure S. Figure 3(b) displays the Si *K*-edge XANES spectra of S-hyperdoped Si samples and the reference undoped Si(100) sample. The spectra are normalized to have the same area in the energy region between 1855 and 1865 eV [not fully shown in the Fig. 3(b)]. The threshold energy and general line-shape of the Si *K*-edge XANES spectra of the S-hyperdoped Si samples are similar to that of the reference Si(100). The characteristic double-peak feature (indicated by two dashed lines, denoted as feature A_2_) of S-hyperdoped Si samples and reference Si(100) above the threshold and its approximate 1.1 eV energy separation are consistent with earlier studies^32^,^33^,^34^,^35^. Hitchcock *et al*.^32^ interpreted the splitting-feature (A_2_) at ~1840 eV at the Si *K*-edge as associated with the excitation from Si 1*s* to the unoccupied Si 3*p* above the conduction-band-minimum (*E*_CBM_), based on crystal symmetry. Clearly, the doublet Si *K*-edge near-edge feature of reference Si(100) arises from long-range ordering. In S-hyperdoped Si samples, a very slight blurring of the doublet as the SF_6_ gas pressure increases was observed, implying the degradation of the long-range order of sample surface and possible formation of amorphous Si^34^,^36^. Si *K*-edge XANES further supports the Si 2*p* XPS and micro-Raman measurements revealing the pressure-induced formation of amorphous Si and/or Si polymorphs on the surface of the S-hyperdoped Si samples. Annealing the 500 Torr sample at 700 °C causes the relatively clear splitting of the feature again, suggesting that the S-hyperdoped Si sample that was annealed at a high temperature exhibited a structurally ordered surface that reverted closely to that of the reference Si(100). The lower panel in Fig. 3(b) shows the difference between the Si *K* near-edge absorption spectra of S-hyperdoped Si samples and reference Si(100). Notably, the overall net intensity of various spectra (obtained by integrating the feature area in the energy range of 1837–1842 eV) is negative, and the intensity of feature A_2_ of the 100 Torr, 500 Torr and annealed samples (that are at 500 °C or 700 °C) is lower than that of reference Si(100), indicating an increase (or decrease) in the number of the occupied (unoccupied) DOSs of Si 3*p* in the conduction band of the S-hyperdoped Si samples relative to that of Si(100). This phenomenon can be associated with the partially S-dopant states within the band gap of Si, located close to *E*_CBM_; these merge with the conduction bands of Si and act as electron donors, as proposed by several studies^4^,^5^,^10^,^11^. Annealing the 500 Torr sample at 700 °C caused the difference in the feature A_2_ almost to disappear, suggesting that annealing the sample to 700 °C made the DOSs of Si 3*p* in the hyperdoped Si very close to that of pure Si(100). Evidently, the fact that Si *K*-edge XANES exhibit variations in the DOSs of Si 3*p* at/above *E*_CBM_ demonstrate that S hyperdoping modifies the electronic structure of Si and create structural disordering in the samples. However, annealing the 500 Torr sample to 700 °C caused the DOSs of the Si 3*p* orbital to revert to one similar to that of reference Si(100). It also indicates that a high temperature annealing reduces structural disorder in S-hyperdoped Si and exhibited a structurally ordered surface that reverted closely to that of the reference undoped Si(100).

Figure 3(c) plots the Fourier transform (FT) of the Si *K*-edge EXAFS spectra, and corresponding *k*^3^χ data (inset) for the S-hyperdoped Si samples and the reference Si(100), further confirming the structural ordering of Si atoms in the S-hyperdoped Si samples. The first main feature in the FT spectra of the S-hyperdoped Si samples (indicated by the first vertical arrow) is at ~2.3 Å and corresponds to the NN Si-Si bond length (without phase correction); this position is almost the same as that of the feature of reference undoped Si(100)^25^,^37^. The second and third main FT feature (also indicated by vertical arrows) at ~3.6 Å and 4.3 Å reveal the high-shell Si-Si bond distances. The general line-shape and radial distribution of the FT spectra of the 100 Torr, 500 Torr and annealed samples (annealed at 500 °C and 700 °C) are close to those of reference Si(100), indicating that the local atomic structures of Si atoms in the S-hyperdoped Si samples are similar to that of reference Si(100). However, the S-hyperdoped Si samples exhibit large structural disorder (or high Debye-Waller factors) around Si atoms, as evidenced by the lower main feature intensities in the FT spectra than those of the reference Si(100). The structural disordering of Si may have been caused by the S-dopant diffusion which increases structural disorder and affects the intensity of the FT-feature of the S-hyperdoped Si samples to be lower than that of reference Si(100). Especially, the intensity of the main FT-features of the sample that was annealed at 700 °C is higher than those of the other S-hyperdoped Si samples. The moderately high intensity of the feature in the FT spectrum of 700 °C sample in Fig. 3(c) suggests that the structural ordering of Si in the annealed sample, resembles closely that of reference Si(100). This result reveals that annealing at 700 °C restores structural ordering close to that of reference Si(100), which finding is consistent with the finding Si *K*-edge XANES spectra that are shown in Fig. 3(b). On the other hand, as discussed by Newman *et al*.^8^, the Se *K*-edge EXAFS revealed that the NN Se-Si bond distance increased with the sub-band gap absorptance. Newman *et al*. also observed that annealing the samples reduces the Se-Si bond length and the sub-band gap absorptance, owing to the formation of the SiSe_2_ precipitate. In Si *K*-edge EXAFS spectra that are shown in Fig. 3(c), no clear change in the Si-Si bond length is observed and the formation of Si-S precipitates in S-hyperdoped Si samples is unlikely. If Si-S precipitates were formed in the S-hyperdoped Si samples, then the extra feature of NN Si-S would have appeared at a bond length of ~2.1 Å in the FT spectrum^10^,^38^. The FT spectra in Fig. 3(c) show no clear changes in the general line-shape, and the splitting of the first main feature (near 2.3 Å) in the FT spectra rules out the possibility that the change of the sub-band gap absorption is directly related to the formation of Si-S precipitates in the S-hyperdoped Si samples. Importantly, the 500 Torr sample has an average IR absorptance of ~87%, but annealing at 500 °C reduces the average IR absorptance to ~23% and annealing at 700 °C reduce it further to ~7%. Both the Si *K*-edge XANES and EXAFS results showed that the sample annealed at the high temperature of 700 °C and the undoped Si(100) reference have similar near-edge absorption feature and structural ordering, illustrating that the sub-band gap absorption of the S-hyperdoped Si samples is clearly related to their electronic states and atomic structural ordering, which are also influenced by the concentration of S-dopant and thermal annealing.

**Figure 4:**
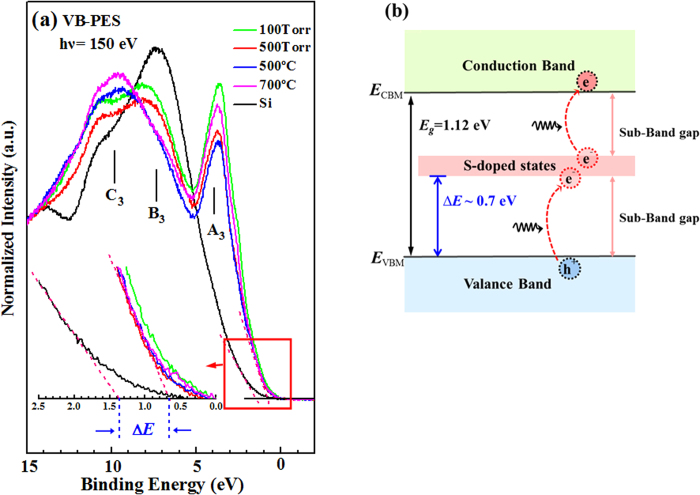
(**a**)VB-PES spectra of hyperdoped Si samples and reference undoped Si(100). (**b**) S dopants introduce electron states or impurity band 0.7 eV above *E*_VBM_ of reference Si. S-dopant states or impurity band located in band gap of Si facilitates generation of charge carriers that participate in absorption of two or more lower-energy photons.

Figure 4(a) displays the VB-PES spectra of S-hyperdoped Si and reference undoped Si(100) samples that were obtained using a photon energy of 150 eV; the spectra include three major features, A_3_-C_3_. The VB-PES spectra of the S-hyperdoped Si samples and reference Si(100) differ strikingly. Strong S-dopant states, feature A_3_ (at ~3.5 eV), were clearly observed in the S-hyperdoped Si samples and the 100 Torr sample had the most intense feature A_3_. Notably, the SEM images of S-hyperdoped Si samples [Fig. 1(a)–(d)] reveal that the surfaces of the S-hyperdoped Si samples are quite rough and S cannot be distributed uniformly over the sample. In the SPEM measurement^39^, the randomly selected region of the surface of the highly S-hyperdoped sample may have contained less S, explaining the non-linear increase of the intensity A_3_ with the concentration of S. Although the 500 Torr sample has the highest concentration of S, it does not have the most intense feature A_3_ in Fig. 4(a). Since the photoionization cross-section of S 3*p* is nearly four times that of Si 3*p* at an excitation photon energy of hν = 150 eV^40^, feature A_3_ of S-hyperdoped Si samples can be assigned mainly to the S-dopant states at/near *E*_VBM_ or below zero energy (corresponding to 0 eV)^41^,^42^,^43^. The reference Si(100) exhibits broad features that are centered at ~7.5 eV and 10 eV below the zero energy denoted as B_3_ and C_3_, respectively, attributed to the Si 3*s*-3*p* and 3*s*-like bands^25^,^43^,^44^,^45^,^46^. Apparently, the *E*_VBM_ of the S-hyperdoped Si samples was closer to zero energy than that of the reference Si(100). Typically, the *E*_VBM_ of the samples is precisely determined by extrapolating the leading edge of the spectrum to the baseline at its largest slope^47^,^48^,^49^,^50^. The magnified region in the lower panel of Fig. 4(a) reveals an *E*_VBM_ of the S-hyperdoped Si samples is at ~0.7 eV, which is 0.7 eV shifted above in relative to that of the reference Si(100) which has E_VBM_ at ~1.4 eV. The shift in the *E*_VBM_ of the S-hyperdoped Si samples from that of reference Si(100) reveals that S dopants induced formation of electron states or an impurity band at ~0.7 eV above the *E*_VBM_ of pure Si as shown in Fig. 4(b)^42^,^51^,^52^, which is schematically depicted as an energy level between the S-dopant states and *E*_VBM_/*E*_CBM_ of Si. Earlier studies have suggested that chalcogen dopants, which can introduce various electron states deep in the band gap of Si, act as electron donors and may form an impurity band^2^,^53^,^54^. Such localized impurities have been accepted as being responsible for non-radiative recombination, in which process a free electron and a free hole recombine in the material. When the impurity band is formed in an adequately high concentration of dopant, and two photons have less energy than the band gap (sub-band gap), a free electron-hole pair is formed via an impurity energy level somewhere in the band gap, as schematically presented in Fig. 4(b), significantly increasing the absorption of IR energy from the solar spectrum^2^,^55^,^56^. Recently, Fabbri *et al*.^57^ found that S-induced luminescence around 0.85 eV which is at an energy that is deep inside the band gap of a substitutional S atom or a charged S dimer in Si; they also observed that the quenching of luminescence correlated closely with predicted overlap between the impurity band and the conduction band of Si. The VB-PES spectra of S-hyperdoped Si samples herein reveal that the S dopants introduced electron states ~0.7 eV above the *E*_VBM_ of pure Si, not only forming an impurity band deep inside the band gap of Si that gives rise to strong sub-band gap absorption, but also causing an IMT when the S dopant is at/above a critical concentration^4^,^5^,^11^. The concentrations of S dopant should be similar to or greater than the Mott transition critical concentration in order to observe metallic-like conduction owing to overlap of the wave-functions of two impurity sites^48^. The critical concentration for a Mott transition to metallic-like conduction for hyperdoped Si samples has been estimated to be near 10^19^ cm^−3^^2^,^3^. Accordingly, in this study, in which the S doping in the Si was estimated to be greater than approximately 10^20^ cm^−3^, the S donors were at/above the critical density for a Mott transition; the induced deep S-dopant states presumably behaved in a manner that was similar to metallic-like conduction owing to a delocalized electron wave-function inside the band gap of Si, and the S-dopant states, assumed to be delocalized states, formed an impurity band, which was responsible for the metallic-like conduction inside the band gap of the hyperdoped Si samples^42^,^51^,^52^.

**Figure 5:**
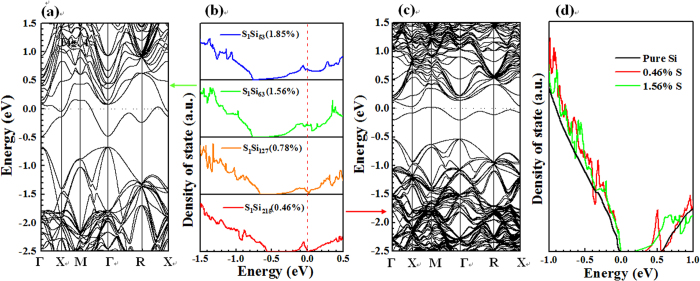
Doping induced IMT in S-hyperdoped Si. (**b**) Normalized total DOSs (referenced to Fermi energy indicated as dashed line) versus S doping concentration (also denoted as S_1_:Si_N−1_). (**a**) and (**c**) Corresponding band structures of S_1_:Si_63_ and Si_1_Si_215_, respectively. (**d**) Calculated normalized DOSs of pure Si (black), 1.56% (green), and 0.46% (red) S doping concentrations (aligned with the *E*_VBM_).

The S dopants introduced electron states, forming an impurity band within the band gap of the hyperdoped Si, and an IMT was observed at a critical concentration of S dopant. Related theoretical calculations were performed by means of the first-principles full-potential projector augmented wave (PAW) method^58^, implemented in the Vienna *ab initio* simulation package (VASP) package^59^. These calculations were based on the DFT method in the generalized gradient approximation with the Perdew–Burke–Ernzerh of (PBE) functional^60^. The plane-wave energy cutoff has been set to 500 eV. The Brillouin zone has been sampled in a 8×8×8 *k*-grid to carry out the total energy calculation and defect-induced structural optimization. The atomic positions have been relaxed until all of the residual forces were converged to better than 0.001 eV/Å^−1^. To simulate various concentrations of S doping in Si, the electronic DOSs for supercells of size *n*×*n*×*n* for a two-atom primitive cell (*n* = 2, 3, 4 and 5) and an eight-atom cubic cell (*n* = 2 and 3) were calculated. This sampling corresponds to systems of S_1_: Si_*N−*1_, for *N* = 16, 54, 64, 128, 216 and 250 in which the defect spacing increases uniformly from one supercell to the next. The calculated total DOSs in Fig. 5(b) and the corresponding band structures in Fig. 5(a) with specific S-dopant concentrations of 1.56% and 0.46%, respectively, show that the S-dopant states gradually merge into the conduction bands as the concentration of S dopant increases. In particular, the significant decrease in the bandwidth of the doping state from ~0.6 eV to 0.2 eV with the reduction of the S-dopant concentration from 1.56% to 0.46% reveals that the localization of S-dopant states is enhanced substantially as the doping concentrations decreases. Indeed, the calculations further predicted the IMT of S-doped Si at a dopant concentration of about 0.46% (~2.3 × 10^20^ cm^−3^). This result is consistent with previous measurements of the IMT in S-doped Si (1.8 × 10^20^ < n < 4.3 × 10^20^ cm^−3^)^4^ although the underestimate of the band gap in the DFT calculation could be improved by performing large-supercell calculations with the advanced hybrid functional [Fig. S4(a) of the Supplementary Information]^61^, G^0^W^0^ approximation^62^ or more sophisticate quantum Monte Carlo (QMC) method^63^, if the highly computational demand can be met. However, a recent investigation on a similar chalcogen-hyperdope Se:Si system^4^ also illustrated such dopant-induced IMT could be a strong Mott-type transition in which DFT calculation provides a reasonable prediction of IMT transition point even though the semi-local functional of PBE may delocalize the defect states. Meanwhile, to compare the VB-PES data with the theoretical data, Fig. 5(d) presents the DOSs that was aligned with *E*_VBM_. The calculated total DOSs are similar to the observed VB-PES data, showing the yield of the S-dopant states near *E*_VBM_. According to a partial DOSs analysis [Fig. S4(b) and S4(c) of the Supplementary Information], the host Si *p* orbital clearly dominates the DOSs near *E*_VBM_. However, a non-negligible contribution by the *p*-orbital of the S dopant is also evident in the region of *E*_VBM_. Like that of Se-hyperdoped Si^5^, the calculated DOSs of S-hyperdoped Si, shown in Fig. 5(a)–(d), clearly indicate that the enhancement of the DOSs at/above (at/below) the *E*_CBM_ (*E*_VBM_) is associated with the S-dopant states that are created by the S hyperdoping of the Si samples. The calculations also reveal the onset of the IMT in S-doped Si samples at a dopant concentration of lower than 0.5%. The XANES spectra, VB-PES data and theoretical calculations demonstrate that the S hyperdoping changes the electronic structure of Si by forming S-dopant states in the band gap of Si, which is closely associated with the sub-band gap absorptance.

Janzén *et al*.^53^ investigated the S-dopant states in Si using high-resolution IR absorption; they identified that the neutral S (S^0^) form states ~0.75 eV above the valence band edge. The shallow S level resides 0.45 eV above the valence band. The other S states have an energy that is close to the conduction band of Si. However, our S-hyperdoped Si absorptance spectra do not show any feature corresponding to sulfur related energy states in the band gap. In the future, we will plan to probe sulfur related deep states in the band gap at low temperature and at far-IR wavelength in an attempt to reduce thermal broadening as well as deep-level transient spectroscopy measurements could be useful to learn more about the specific transition energy for infrared absorption^3^,^12^.

Based on experimental and theoretical studies above, we have proposed explanation for the sub-band gap absorptance in the S-hyperdoped Si samples. In the present study, the observed changes in the sub-band gap absorptance of the S-hyperdoped Si samples were clearly related to the chemical state of the S species and the S-dopant states in the band gap of Si. The XPS study indicated that the maximum (~87%) sub-band gap absorbing sample (500 Torr sample) contains a high concentration of S, and especially S^2−^ species. Annealing the 500 Torr sample at 700 °C forms large clusters of S_n_^2−^ (n > 2) and reduces the sub-band gap absorpatnce to ~7%. According to the VB-PES spectra and theoretical calculations, the S-dopant forms an impurity band at ~0.7 eV above the *E*_VBM_ of pure Si(100), giving rise to the sub-band gap absorptance that was measured herein. Despite the fact that the larger S clusters (S_n_^2−^, n > 2) may also form deep levels above the valence band, the high concentration of larger S clusters (S_n_^2−^, n > 2) in the annealed samples may have been responsible for the drop in the sub-band absorptance^5^. Again, the overall integrated intensity of feature A_2_ is negative [as presented in the lower panel of Fig. 3(b)], indicating that the increase (decrease) in the DOSs of occupied (unoccupied) Si 3*p* orbitals is related to the S-dopant states, because these merge with the conduction band and act as electron donors in the S-hyperdoped Si samples.

In summary, the formation of S-dopant states in the band gap of Si that contains at/above critical concentration of S leads to high sub-band gap absorption in S-hyperdoped Si samples. The annealing of S-hyperdoped Si sample at 700 °C returns back to the electronic and atomic structures as that of reference Si(100), and cause S species to form larger clusters (S_n_^2−^, n > 2), reducing the sub-band gap absorption. Our theoretical calculations based on the DFT method are consistent with experimental results and the IMT of S-doped Si is predicted to occur at an S concentration at a critical value of 0.46% (~2.3 × 10^20^ cm^−3^).

## Methods

### Preparation of S hyperdoped Si

Si can typically be hyperdoped with S by either ion implantation plus pulse laser annealing or fs-laser doping^2^,^14^. One advantage of fs-laser doping is that the process can roughen the material surface, increasing its absorption of light. S-hyperdoped Si is fabricated by irradiation of Ti: sapphire laser pulses (70 fs) in the presence of SF_6_, which is a precursor of the S as dopant. The *p*-type Si(100) wafer has high resistivity (3–10 kΩ · cm) to minimize other effects of dopants. Before irradiation, the wafer was cleaned by the RCA (Radio Corporation of America) method to remove possible contamination from the surface. The laser pulses were focused to 150 μm and the surface was raster-scanned at 8 kJ/m^2^ and 50 shots/area. Each sample had a size of 1 cm^2^. The SF_6_ pressure was varied to fabricate hyperdoped Si samples with a range of concentrations of S^13^,^15^. The concentration of S-hyperdoped in the Si samples was estimated to be approximately 10^20^ cm^−3^.^2^,^3^ Two pressures (100 Torr and 500 Torr) were used to achieve a difference in S doping level of about one order of magnitude (The concentration of S in Si samples was verified by Rutherford backscattering spectroscopy and secondary ion mass spectroscopy)^2^. The 500 Torr sample was respectively annealed at 500 °C and 700 °C for 30 min. to elucidate the effect of annealing on the chemical states and electronic and atomic structures of both the S dopant and the Si host, and to investigate further their correlations with the sub-band gap absorption in the hyperdoped Si samples.

### Synchrotron-related measurements and characterizations

S *K*-edge XANES, Si *K*-edge XANES, EXAFS and VB-PES experiments were performed at the 16A- and 09A-beamline, at the National Synchrotron Radiation Research Center (NSRRC) in Hsinchu, Taiwan. The XANES and EXAFS measurements at the S and Si *K*-edge were performed in total electron yield modes. The VB-PES spectra were obtained by SPEM. The SPEM measurements were made using a flexure scanning stage and a hemispherical electron-analyzer system. A hemispherical analyzer with a 16-channel multichannel detector was used to collect photoelectrons. The VB-PES spectra were calibrated with reference to the zero energy of clean gold metal, and the energy of the incident X-ray was fixed at 150 eV with the energy resolution set to better than 0.1 eV. The zero energy is the threshold of the emission spectrum. The S and Si core-level XPS spectra of hyperdoped Si samples were also obtained at the 09A-beamline at NSRRC. Before the synchrotron-related measurements were conducted, hydrofluoric (HF) acid solution (1%) was utilized to eliminate the possibility of the presence of native oxides on the sample surface, and the samples were immediately transferred to a vacuum chamber. The core-level XPS spectra were feature-fitted using XPSPEAK41 software after a Shirley-type background had been subtracted from them. The IR absorption of hyperdoped Si samples was measured using an ultraviolet-visible-near IR spectrophotometer that was equipped with an integrating sphere to calculate the absorptance, A = 1-R-T (R = reflectance and T = transmittance). The surface morphology of the hyperdoped Si samples was also characterized using a field-emission SEM.

## Additional Information

**How to cite this article**: Limaye, M. V. *et al*. Understanding of sub-band gap absorption of femtosecond-laser sulfur hyperdoped silicon using synchrotron-based techniques. *Sci. Rep*. **5**, 11466; doi: 10.1038/srep11466 (2015).

## Supplementary Material

Supplementary Information
