# Supplementary material for: Understanding of sub-band gap absorption of femtosecond-laser sulfur hyperdoped silicon using synchrotron-based techniques
Source: Sci Rep. 2015 Jun 22;5:11466. doi: 10.1038/srep11466 (PMC4476416; doi:10.1038/srep11466)
Supplement: Supplementary Information [file srep11466-s1.doc]

**Supplementary Information**

**Understanding of sub-band gap absorption of femtosecond-laser sulfur hyperdoped silicon using synchrotron-based techniques**

Mukta V. Limaye^1,2,†^, S. C. Chen^1,†^, C. Y. Lee^1^, L. Y. Chen^1^, Shashi B. Singh^1,2^, Y. C. Shao^1^, Y. F. Wang^1^, S. H. Hsieh^1^, H. C. Hsueh^1,*^, J. W. Chiou^3^, C. H. Chen^4^, L. Y. Jang^4^, C. L. Cheng^5^, W. F. Pong^1,*^, Y. F. Hu^6^

*^1^Department of Physics, Tamkang University, Tamsui 251, Taiwan*

*^2^Department of Physics, Indian Institute of Science Education and Research, Bhopal 462066, India*

*^3^ Department of Applied Physics, National University of Kaohsiung, Kaohsiung 811, Taiwan*

*^4^ National Synchrotron Radiation Research Center, Hsinchu 300, Taiwan*

*^5^ Department of Physics, National Dong Hwa University, Hualien 974, Taiwan*

*^6^ Canadian Light Source Inc., Saskatoon SK S7N OX4, Canada*

| **Samples** | **Oxidation States** | **Peak B.E.** | **% of components** | **Atomic %** | **Total at. %** | **FWHM (eV)** |
| --- | --- | --- | --- | --- | --- | --- |
| **Sulfur** | **Monosulfide (S^2-^)** | **161.26** | **12.1** |  |  | **0.96** |
|  | **Disulfide (S_2_^2-^)** | **162.30** | **34.3** |  |  | **1.15** |
|  | **Polysulfides (S_n_^2-^, n> 2)** | **163.30** | **29.5** |  |  | **1.09** |
|  | **Elemental Sulfur (S^0^)** | **164.44** | **24.1** |  |  | **1.00** |
| **100 Torr** | **Monosulfide (S^2-^)** | **161.76** | **9.1** | **0.09** |  | **1.02** |
|  | **Disulfide (S_2_^2-^)** | **162.42** | **15.4** | **0.15** |  | **0.85** |
|  | **Polysulfides (S_n_^2-^, n> 2)** | **163.51** | **36.0** | **0.34** |  | **1.52** |
|  | **Elemental Sulfur (S^0^)** | **164.80** | **15.0** | **0.14** |  | **1.50** |
|  | **Sulfite (SO_3_^2-^) /Sulfate (SO_4_^2-^)** | **168.58** | **24.5** | **0.23** | **0.95** | **4.15** |
| **500 Torr** | **Monosulfide (S^2-^)** | **161.69** | **15.1** | **0.19** |  | **1.45** |
|  | **Disulfide (S_2_^2-^)** | **162.60** | **21.8** | **0.28** |  | **1.58** |
|  | **Polysulfides (S_n_^2-^, n> 2)** | **163.53** | **24.6** | **0.31** |  | **1.50** |
|  | **Elemental Sulfur (S^0^)** | **164.75** | **17.3** | **0.22** |  | **1.62** |
|  | **Sulfite (SO_3_^2-^) /Sulfate (SO_4_^2-^)** | **168.63** | **21.2** | **0.27** | **1.27** | **4.84** |
| **500^o^C** | **Monosulfide (S^2-^)** | **161.77** | **4.6** | **0.03** |  | **1.04** |
|  | **Disulfide (S_2_^2-^)** | **162.43** | **20.8** | **0.14** |  | **0.89** |
|  | **Polysulfides (S_n_^2-^, n> 2)** | **163.48** | **34.5** | **0.23** |  | **1.18** |
|  | **Elemental Sulfur (S^0^)** | **164.51** | **6.2** | **0.04** |  | **1.81** |
|  | **Sulfite (SO_3_^2-^) /Sulfate (SO_4_^2-^)** | **168.53** | **33.9** | **0.23** | **0.67** | **4.35** |
| **700^o^C** | **Monosulfide (S^2-^)** | **161.72** | **2.2** | **0.01** |  | **0.84** |
|  | **Disulfide (S_2_^2-^)** | **162.47** | **22.7** | **0.05** |  | **0.87** |
|  | **Polysulfides (S_n_^2-^, n> 2)** | **163.54** | **36.6** | **0.07** |  | **1.28** |
|  | **Elemental Sulfur (S^0^)** | **164.66** | **8.7** | **0.02** |  | **1.49** |
|  | **Sulfite (SO_3_^2-^) /Sulfate (SO_4_^2-^)** | **168.74** | **29.8** | **0.06** | **0.20** | **4.47** |

**Table SI.** Detailed parameters like oxidation states, peak position, atomic % of each component of the S in hyperdoped Si, total at.% and full width at half maximum (FWHM) of samples obtained by the deconvolution of the S 2*p* XPS curves.

**Figure S1.** XPS survey scan of hyperdoped Si samples along with pure Si(100) and reference S.

The hyperdoped Si samples show the presence of S in all the four samples. It indicates that the S is doped in all the four samples. The atomic percentage of S in each sample is tabulated in Table SII.

**Table S II.** Analysis of survey scan of hyperdoped Si samples. Pure Si (100) and S are used for comparison.

| **Sample** | **C (2*p*)** | **N (1*s*)** | **O (1*s*)** | **Si (2*p*;1*s*)** | **Si (2*p*;2*s*)** |
| --- | --- | --- | --- | --- | --- |
| **Si** | **26.03%** | **2.09%** | **23.80%** | **32.60% (2*p*)**  **15.48% (1*s*)** |  |
| **S** | **32.73%** |  | **26.93%** |  | **24.56% (2*p*)**  **15.78% (2*s*)** |
| **100 Torr** | **18.04%** | **2.60%** | **35.76%** | **35.87% (2*p*)**  **6.78% (1*s*)** | **0.95% (2*p*)** |
| **500 Torr** | **19.82%** |  | **46.23%** | **30.52% (2*p*)**  **2.16% (1*s*)** | **1.27% (2*p*)** |
| **500^o^C** | **12.63%** |  | **36.18%** | **43.56% (2*p*)**  **6.96% (1*s*)** | **0.67% (2*p*)** |
| **700^o^C** | **16.88%** |  | **34.94%** | **41.48% (2*p*)**  **6.50% (1*s*)** | **0.20% (2*p*)** |

**Figure S2**. Core level XPS spectra of Si 2*p* of hyperdoped Si samples 100Torr, 500Torr, 500^0^C and 700^0^C. For comparison the standard samples pure Si(100) is also shown in the plot.

Figure S2 presents the Si 2*p* core level XPS spectra of hyperdoped Si samples that were prepared at 100 Torr and 500 Torr, and of 500 Torr samples that were annealed at 500^0^C or 700^0^C. The spectrum of pure Si(100) is shown for comparison. The deconvoluted Si 2*p* core level XPS spectra had five peaks. The peaks for Si 2*p* were at binding energies of 99.6 eV for Si 2*p*_3/2_ (Si^0^) , 100.2 eV for Si 2*p*_1/2_ (Si^0^), 100.6 eV for Si^1+^, 102.7 eV for Si^3+^, and 103.8 eV for Si^4+^.^1-3^ Table SIII (Supplementary information) presents in detail the parameters of the deconvoluted core-level spectra of all four samples, including the positions and FWHM of the peaks. Spin-orbit splitting of 0.6 eV was observed between Si 2*p*_3/2_ and Si 2*p*_1/2_.^1,4^ The peaks at 100.6 eV, 102.7 eV and 103.8 eV arise from the different oxygen-containing silicon species Si_2_O, Si_2_O_3_ and SiO_2_, respectively.^5^

| **Sample** | **Oxidation states** | **Peak B.E. (eV)** | **% of components** | **FWHM (eV)** |
| --- | --- | --- | --- | --- |
| **Si** | **Si^0^ (2p_3/2_)** | **99.6** | **56.9** | **1.12** |
|  | **Si^0^ (2p_1/2_)** | **100.2** | **31.6** | **1.23** |
|  | **Si^1+^** | **100.6** | **3.7** | **0.95** |
|  | **Si^3+^** | **102.6** | **4.4** | **1.34** |
|  | **Si^4+^** | **103.4** | **3.4** | **1.28** |
| **100 Torr** | **Si^0^ (2p_3/2_)** | **99.3** | **64.2** | **1.50** |
|  | **Si^0^ (2p_1/2_)** | **100.1** | **20.7** | **1.22** |
|  | **Si^1+^** | **100.9** | **8.8** | **1.53** |
|  | **Si^3+^** | **102.4** | **4.4** | **1.62** |
|  | **Si^4+^** | **103.4** | **1.9** | **1.27** |
| **500 Torr** | **Si^0^ (2p_3/2_)** | **99.4** | **60.0** | **1.53** |
|  | **Si^0^ (2p_1/2_)** | **100.0** | **25.7** | **1.39** |
|  | **Si^1+^** | **100.6** | **8.4** | **1.18** |
|  | **Si^3+^** | **102.7** | **4.5** | **1.38** |
|  | **Si^4+^** | **103.3** | **1.4** | **1.10** |
| **500^o^C** | **Si^0^ (2p_3/2_)** | **99.6** | **50.0** | **1.25** |
|  | **Si^0^ (2p_1/2_)** | **100.1** | **37.7** | **1.28** |
|  | **Si^1+^** | **100.7** | **7.1** | **1.04** |
|  | **Si^3+^** | **102.7** | **3.0** | **1.49** |
|  | **Si^4+^** | **103.6** | **2.2** | **1.34** |
| **700^o^C** | **Si^0^ (2p_3/2_)** | **99.5** | **59.2** | **1.46** |
|  | **Si^0^ (2p_1/2_)** | **100.2** | **33.7** | **1.38** |
|  | **Si^1+^** | **100.8** | **3.1** | **0.94** |
|  | **Si^3+^** | **102.9** | **3.5** | **1.48** |
|  | **Si^4+^** | **103.7** | **0.5** | **0.99** |

**Table SIII.** Detailed parameters like oxidation states, peak position, full width at half maximum (FWHM), and % of each component of the S in hyperdoped Si samples obtained by the deconvolution of the Si 2*p* XPS curves.

**Figure S3.** Raman spectra of hyperdoped Si samples along with pure Si(100) as a reference. The inset plot the magnified Raman spectra in the range of 250-500 cm^-1^.

The micro-Raman spectra of hyperdoped Si samples, 100 Torr and 500 Torr, show the pressure-induced formation of crystalline silicon polymorphs Si-III and Si-XII under their surfaces.^6-8^ The observed Raman modes were attributed to Si-XII (354 cm^-1^, 395 cm^-1^), Si-III (387 cm^-1^, 443 cm^-1^) and Si-I (broad peak at 300 cm^-1^). The intensities of the Si polymorph Raman modes are lower than that of the Si-I peak at 519 cm^-1^, suggesting that a smaller volume of Si polymorphs is generated than in Si-I. Two characteristic peaks, Si-XII (354 cm^-1^, 395 cm^-1^) and Si-III (387 cm^-1^, 443 cm^-1^), were observed.^6-8^

The formation of metastable Si polymorphic phases may arise from the light-matter interactions that occur during the fs-laser doping, which produces pressure waves that induce phase transformations in Si.^6^ However, their exact mechanism of formation of polymorphis phase and its influence on the doping process are unknown.^6^ Previous studies have shown that Si is transformed from its diamond cubic phase (Si-I) to a *β*-Sn phase (Si-II) at pressures above ~12 GPa and that this transformation is irreversible, meaning that Si-II cannot transform back to Si-I during reducing the pressure.^6^ A sufficiently slow release of pressure will cause Si with an R8 structure (Si-XII) to nucleate from Si-II and then to transform reversibly to a BC8 structure (Si-III) at around 2 GPa.^6^ Annealing the 500 Torr sample at 500^o^C or 700^o^C caused the polymorph features to disappear and the Raman spectra to become similar to that of pure Si(100).

**
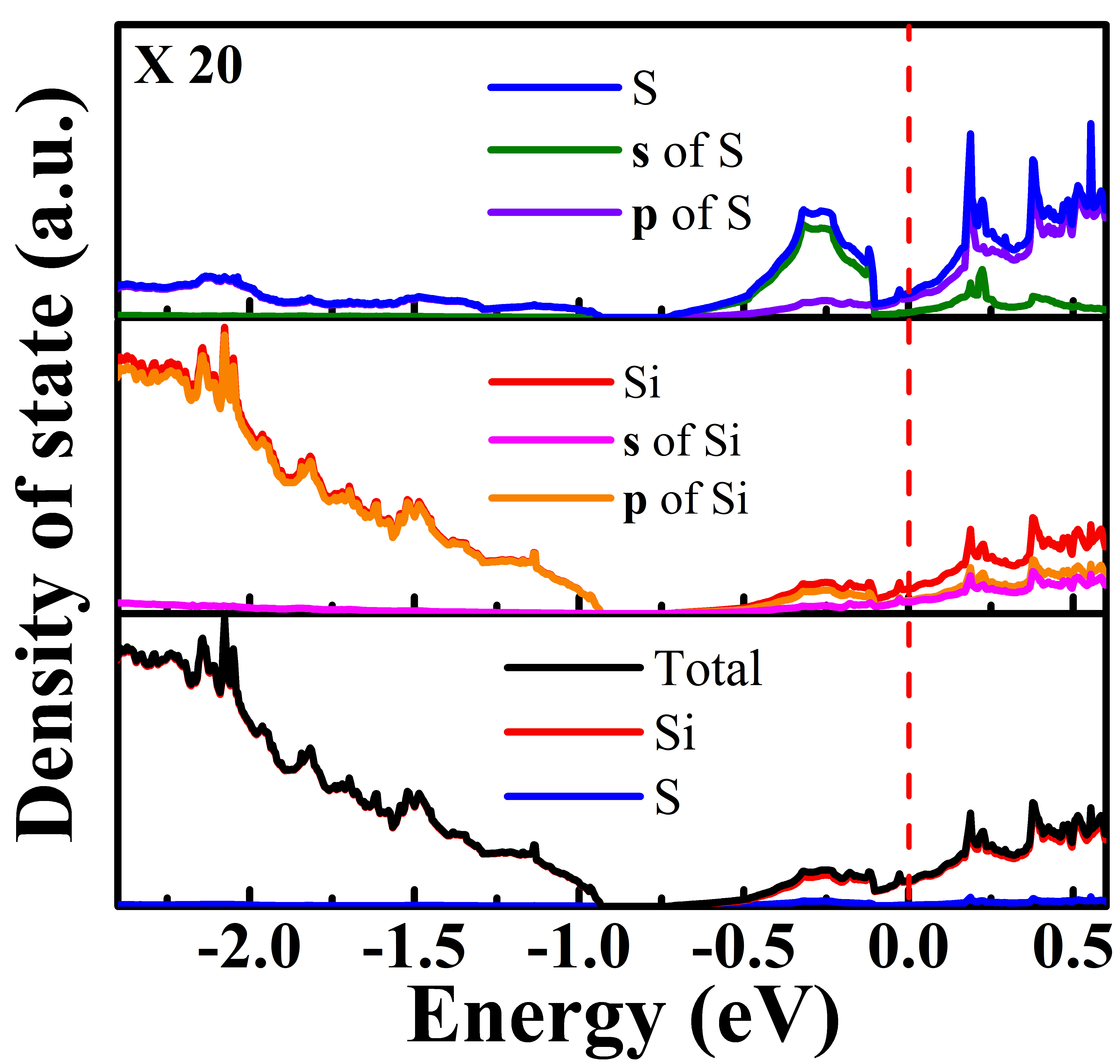

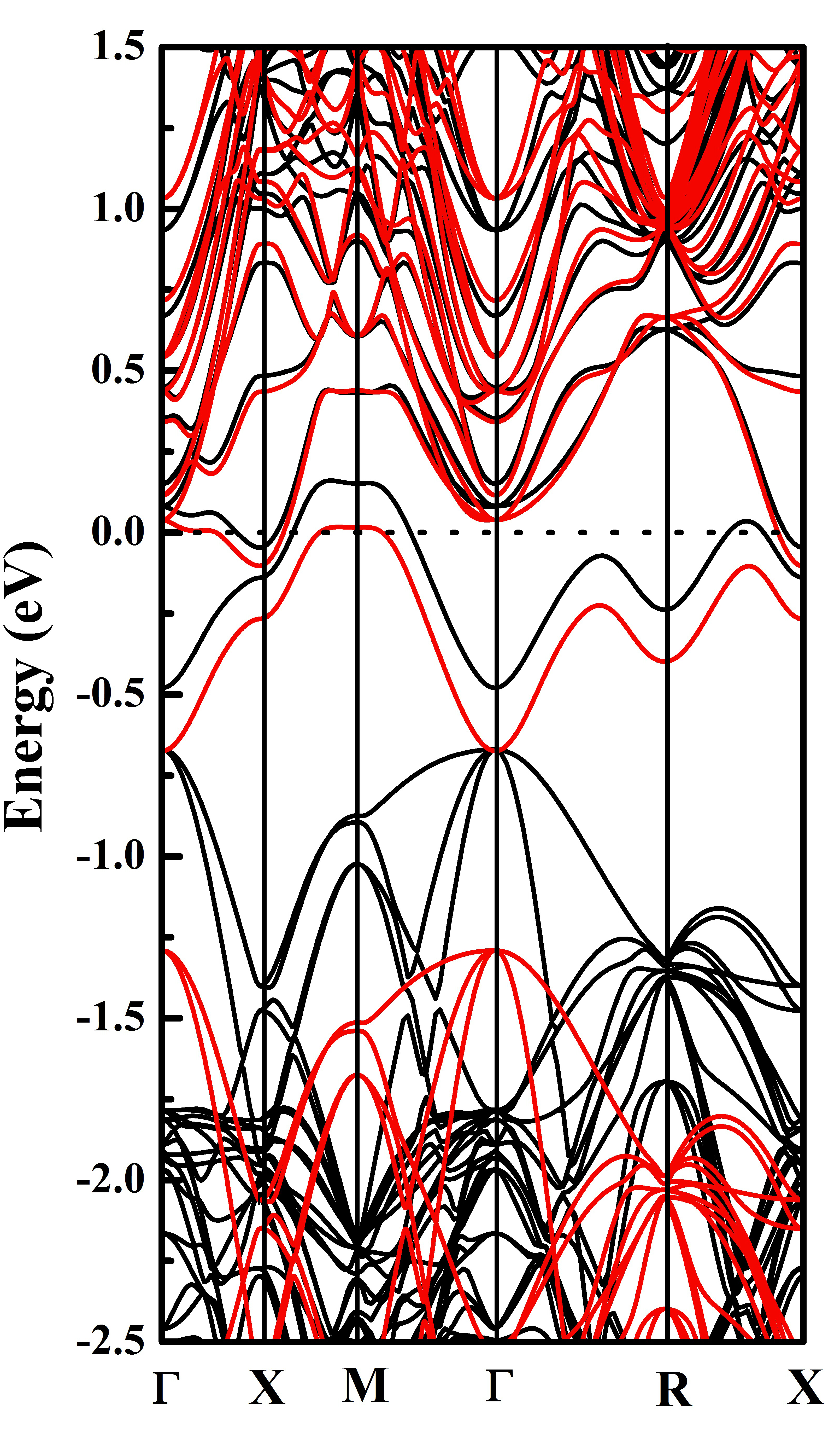
**

**(a)**

**(b)**

**(c)**

**
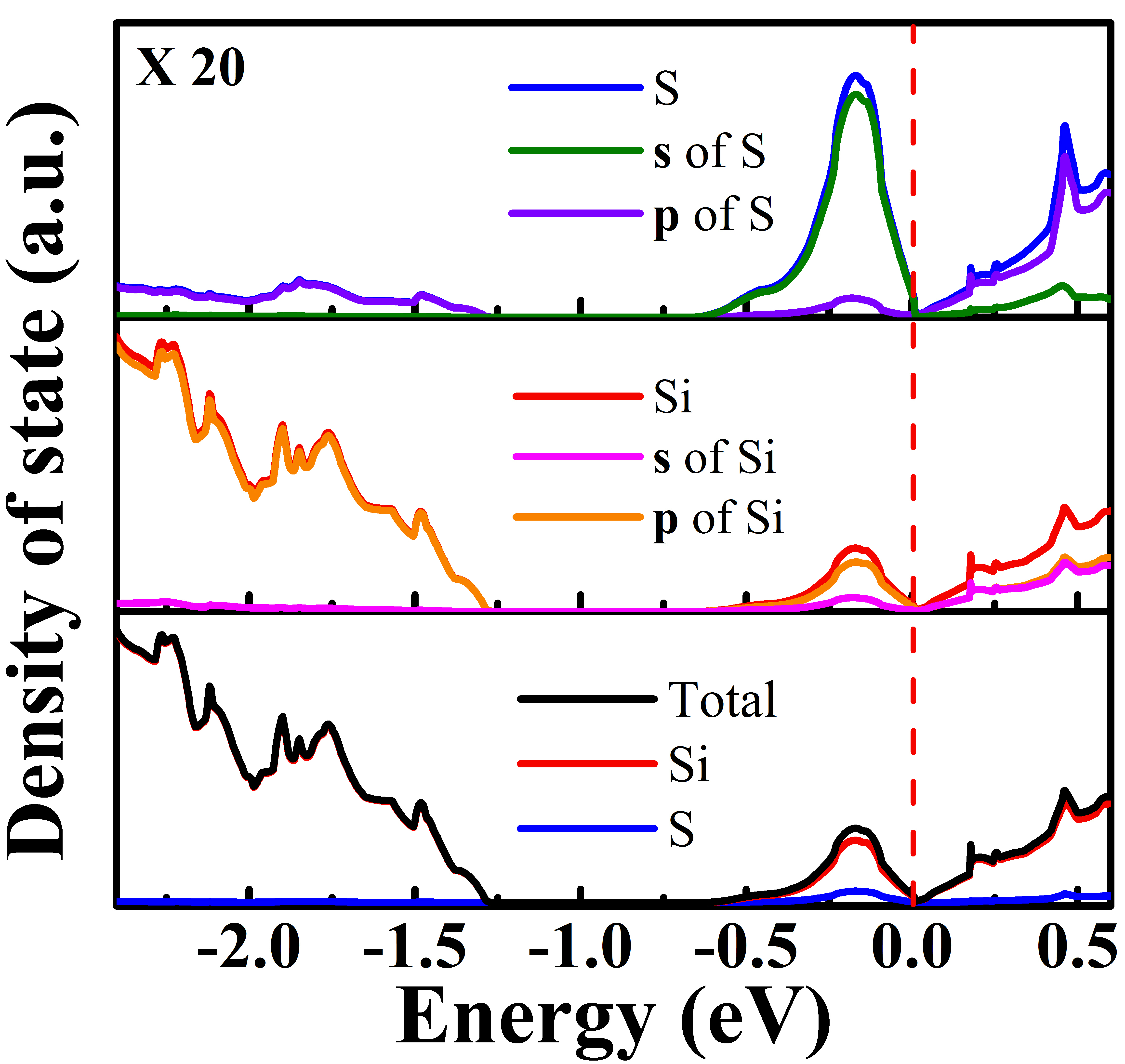
**

**Figure S4.** Electronic band structures (a) and PDOS (b), (c) of S-hyperdoped Si (S_1_:Si_63_), in a 2*×*2*×*2 supercell of the conventional 8-atom cubic cell, calculated in PBE functional [black curves in (a) and three panels in (b)] and HSE06 functional [red curves in (a) and three panels in (c)], respectively. The Fermi level is indicated as the dashed lines at zero energy for alignment. The PDOS of dopant S [top panel of both (b) and (c)] are scaled by a factor of 20 for clarity.

Based on the optimized defect structure from PBE calculation, an indirect band gap of 1.18 eV [the energy difference between the VBM at Γ and CBM at X in Fig. S4(a)] calculated by HSE06 method [red curves in Fig. S4(a)] is much more closer to experiment (1.12 eV) than PBE result (0.63 eV). However, HSE06 calculated bandwidth (0.69 eV) of the dispersive defect band megering with conduction bands of S_1_:Si_63_ is in good agreement with PBE calculation (0.64 eV). Furthermore, PDOS analysis of both PBE [Fig. S4(b)] and HSE06 [Fig. S4(c)] calculation show clearly that the defect state in S-hyperdoped Si is dominated by the hybridization of *s*-orbital of the dopant S and *p*-orbital of the neighboring Si atoms.

**References**

1. Meyer, B. Elemental sulfur. *Chem. Rev.* **76**, 367–388 (1976).

2. Khan, S. A. UV-ATR Spectroscopy study of the speciation in aqueous polysulfide electrolyte solutions. *Int. J. Electrochem. Sci.* **7**, 561–568 (2012).

3. Zhang, Y. F., Liao, L. S., Chan, W. H., Lee, S. T. & Sham, T. K. Electronic structure of silicon nanowires: A photoemission and x-ray absorption study. *Phys. Rev. B* **61**, 8298–8305 (2000).

4. Park, C.-M. *et al.* Characterizations and electrochemical behaviors of disproportionated SiO and its composite for rechargeable Li-ion batteries. *J. Mater. Chem.* **20**, 4854 (2010).

5. López, J. A. L. *et al.* Morphological, compositional, structural, and optical properties of Si-nc embedded in SiOx films. *Nanoscale Res. Lett.* **7**, 604 (2012).

6. Smith, M. J.; Lin, Y.-T.; Sher, M.-J.; Winkler, M. T,; Mazur, E.; Gradečak, S. Pressure- induced phase transformation during femtosecond-laser doping of silicon. *J. Appl. Phys.* **110**, 053524 (2011).

7. Smith, M. J. *et al.* The origins of pressure-induced phase transformations during the surface texturing of silicon using femtosecond laser irradiation. *J. Appl. Phys.* **112**, 083518 (2012).

8. Domnich, V.; Gogotsi, Y. Phase transformation in silicon under contact loading. *Rev. Adv. Mater. Sci.* **3**, 1 (2002).
